# Efficacy and Safety of Praziquantel for Treatment of *Schistosoma mansoni* Infection among School Children in Tanzania

**DOI:** 10.3390/pathogens9010028

**Published:** 2019-12-27

**Authors:** Rajabu Hussein Mnkugwe, Omary S. Minzi, Safari M. Kinung’hi, Appolinary A. Kamuhabwa, Eleni Aklillu

**Affiliations:** 1 Department of Clinical Pharmacology, School of Medicine, Muhimbili University of Health and Allied Sciences, Dar es Salaam 11103, Tanzania; rajabuhussein06@gmail.com; 2 Division of Clinical Pharmacology, Department of Laboratory Medicine, Karolinska University Hospital-Huddinge, Karolinska Institutet, 141 86 Stockholm, Sweden; 3 Department of Clinical Pharmacy and Pharmacology, School of Pharmacy, Muhimbili University of Health and Allied Sciences, Dar es Salaam 11103, Tanzania; minziobejayesu@gmail.com (O.S.M.); enali2012@gmail.com (A.A.K.); 4 National Institute for Medical Research (NIMR), Mwanza Research Centre, Mwanza 33104, Tanzania; kinunghi_csm@hotmail.com

**Keywords:** intestinal schistosomiasis, praziquantel, single-dose, efficacy, safety, Tanzania

## Abstract

Single-dose targeted praziquantel preventive chemotherapy is the WHO-recommended intervention for schistosomiasis control in endemic countries. The objective of this study was to assess the efficacy and safety of single-dose praziquantel among *Schistosoma mansoni*-infected children in north-western Tanzania. A prospective safety and efficacy surveillance study was conducted among 341 school-going children treated with a single-dose praziquantel 40 mg/kg body weight. Socio-demographic, pre-treatment, and post-treatment stool examination and safety data were collected. The primary and secondary outcomes were treatment efficacy (parasitological cure and egg reduction rates at three weeks post-treatment) and treatment-related adverse events, respectively. The overall cure rate and egg reduction rate were 81.2% (76.8–85.3%) and 95.0% (92.7–97.3%), respectively. There was no significant association between cure rate and pre-treatment infection intensity. The incidence of treatment-associated adverse events was 28.5% (23.7–33.3%), with abdominal pain being the most common. Post-treatment abdominal pain and vomiting were significantly associated with pre-treatment infection intensity (*p* < 0.001) and anemia (*p* = 0.03), respectively. Praziquantel single-dose is still safe and efficacious against *Schistosoma mansoni* infection. However, the lack of cure in about one-fifth and adverse events in a quarter, of the infected children indicate the need for close praziquantel safety monitoring and treatment optimization research to improve efficacy.

## 1. Introduction

Schistosomiasis is a treatable and preventable neglected tropical disease (NTD) which continues to affect the health and lives of many people, especially children in Sub-Saharan Africa (SSA) [1,2]. The disease is among parasitic diseases of public health importance globally and particularly in Sub-Saharan African countries, including Tanzania [1]. Globally, more than 779 million people are at risk of schistosomiasis infection, and more than 240 million people are infected [3,4,5,6]. More than 90% of the global burden of schistosomiasis is contributed by cases in SSA [7]. Despite being a treatable and preventable disease, schistosomiasis still causes up to 280,000 deaths annually in SSA [4,8]. In Tanzania, both urogenital schistosomiasis (*Schistosoma haematobium*) and intestinal schistosomiasis (*Schistosoma mansoni*) are endemic throughout the country, with the latter being highly prevalent around the Lakes Zones, where a prevalence of up to 100% have been reported [9]. 

The World Health Organization (WHO) recommended large-scale annual or biannual mass drug administration (MDA) of praziquantel to people at risk of the disease, especially school-going children in endemic settings as preventive chemotherapy [10]. To date, praziquantel given as a single dose (40 mg/kg body weight) remains the only efficacious and safe drug recommended by the WHO for the treatment and control of all schistosome species worldwide [3]. However, long-term and repeated use of praziquantel in MDA programs may cause parasite tolerance and a threat of drug resistance. Currently, no confirmed evidence of praziquantel resistance has been documented, but findings of drug tolerance and low cure rates have been reported in field studies from SSA [11]. There is still debate on which term is appropriate, i.e., drug tolerance or drug resistance [12]. Previous reports have indicated the need for regular monitoring of the safety and efficacy of praziquantel [13], as currently; there is no alternative drug for the treatment and control of this poverty-related disease. At the moment, there is no approved vaccine on the market for the prevention of schistosomiasis, although clinical trials are ongoing [14,15]. It is most likely that annual or biannual single-dose praziquantel MDA will remain the mainstay strategy for the treatment and control of schistosomiasis in the immediate future.

The few published studies to have investigated the efficacy and tolerability of praziquantel, especially among school children, have applied varying methods and shown varying results. Most studies used parasitological cure rates to report the drug efficacy, and a few studies have reported efficacy using both parasitological cure rate and egg reduction rate [16]. The use of both parasitological cure rate and egg reduction rate is important, in order for a standardized comparison to be made between studies from different regions [16]. Studies conducted to assess praziquantel efficacy as a single dose of 40 mg/kg body weight reported contradictory findings in terms of parasitological cure rate and egg reduction rate. Some studies have reported a higher cure rates and egg reduction rates with a single dose of praziquantel [17,18,19], while the outcomes were low in other studies [20,21,22]. These inconsistent findings indicate the need for additional studies to generate evidence-based information for policymakers and authorities, such as national NTD control programs. Moreover, single-dose praziquantel has been reported to have different treatment efficacy against different *Schistosoma* species. Praziquantel has been reported to be more efficacious against *S. haematobium* [23] than *S. mansoni* [22]. Due to this observation, close monitoring of drug efficacy in *S. mansoni* infection should be a priority in settings where both species are endemic, like in the Lake Zone in Tanzania. 

Previous studies have reported that single-dose praziquantel at 40 mg/kg body weight continues to be a safe and tolerable drug, especially among school children [24,25]. The most commonly reported adverse events include abdominal pain, vomiting, dizziness, diarrhea, and allergic reactions [25,26], and they are reported to be associated with pre-treatment infection intensity [21]. Heavily-infected patients encounter more and sometimes severe adverse events compared to those with mild to moderate infections [25]. The type and frequency of treatment-associated adverse events vary between populations partly due to genetic differences, environmental factors, and other factors, including nutritional status and disease endemicity. Therefore, it is imperative to monitor the safety and efficacy of praziquantel in various populations and geographic locations, particularly in endemic areas such as the Lake Zone of Tanzania, where the prevalence of *Schistosoma mansoni* is still high, despite repeated preventive chemotherapy [7,9,27,28]. With the current wide implementation of large-scale preventive chemotherapy intervention for the control of NTDs such as schistosomiasis, the WHO recommends assuring drug safety [29] and assessing drug efficacy against schistosomiasis [30]. Therefore, the current study investigated the safety and efficacy of single-dose praziquantel against *Schistosoma mansoni* infection among school children in a rural setting of north-western Tanzania. 

## 2. Results

### 2.1. Socio-Demographic and Baseline Characteristics of the Studied Population

A total of 341 school children infected with intestinal schistosomiasis were enrolled in this study. The mean age of the studied population was 11.8 ± 1.7 years. The majority of the study participants were females (53.1%). At enrolment, about one-fifth (20.8%) of the study participants reported a history of pre-treatment abdominal pain. Before treatment, most of the study participants had moderate infection intensity (44.6%). The mean ± standard deviation of egg count/gram of stool was 365.1 ± 437.4, and the median was 222 (IQR 96–471). Stunting and wasting were 34.3% (95% CI = 29.2–39.5%) and 10.0% (95% CI = 6.8–13.2%), respectively. The median hemoglobin concentration was 12.7 g/dL (IQR 11.6–13. 5) (Table 1).

### 2.2. Cure Rate and Egg Reduction Rate

Assessment of praziquantel for the treatment of schistosomiasis was done using cure rate (CR) and egg reduction rate (ERR) following the WHO guideline [30]. The CR was defined as the proportion of treated persons who were egg-positive at baseline but became egg-negative three weeks after baseline treatment. Of the 341 study participants, 277 (81.2%, 95% CI = 76.8–85.3%) achieved parasitological cure. Though not statistically significant (*p* = 0.79), adolescents (>12 years) were more cured (82.1%) compared to pre-adolescents (80.9%). There was no statistically significant difference in CRs between females and males (*p* = 0.58). Pre-treatment infection intensity (*p* = 0.51), stunting (*p* = 0.39), wasting (*p* = 0.27), and anemia (*p* = 0.07) were not significantly associated with parasitological cure (Table 2). The proportions of the participants with intestinal schistosomiasis infection across all levels of infection intensities after treatment were significantly lower compared to those before treatment in the studied populations (*p* < 0.0001) (Table 3). Variables with a *p*-value of < 0.3 (age, anemia, height, and baseline infection intensity) on univariate regression analysis were included in the multivariate regression model. All variables were not significant predictors of cure (*p* > 0.05). The Hosmer and Lemeshow test for the goodness of fit for multivariate analysis model was a good fit (*χ^2^* = 5.42, *p* = 0.71) (Table 4).

The overall ERR was found to be 95.0% (95% CI = 92.7%–97.3%). Though not significant, the ERR was higher in the adolescent age group (97.6%) compared to the pre-adolescent age group (93.6%) (*p* = 0.12). Males were found to have relatively higher ERR (96.0%) compared to females (94.2%) (*p* = 0.45). Table 5 presents arithmetic means of the egg count at baseline, follow up visit, and ERRs stratified by age group and sex.

### 2.3. Treatment-Associated Adverse Events 

Overall, 97 (28.7%, 95% CI = 23.7–33.3%) of the study participants experienced adverse events within four hours of post-praziquantel administration. A total of 91 (26.7%) participants experienced post-treatment abdominal pain, and 6 (1.8%) participants vomited (two hours post-drug administration). No other gastrointestinal symptoms, including nausea and diarrhea, were observed. No neurological symptoms such as dizziness, fainting, headache were observed. Almost all observed post-treatment abdominal pain and vomiting events were mild and resolved within four hours of drug administration. There was no statistically significant association between CR and post-treatment abdominal pain (*p* = 0.36) or vomiting (*p* = 0.31). The observed post-treatment abdominal pain was significantly associated with pre-treatment infection intensity (*p* < 0.001), but not with age (*p* = 0.16) or sex of the study participant (*p* = 0.25). Of those who reported abdominal pain, most had either moderate infection (25.7%) or heavy infection (40.2%), and only 12.6% had a light infection. Vomiting was significantly associated with anemia (*p* = 0.03) but not with age (*p* = 0.18) or sex (*p* = 0.69). Participants who were anemic experienced vomiting more (5.2%) compared to those without anemia (0.8%) (Table 6).

## 3. Discussion

We conducted an observational safety and efficacy surveillance study of a single-dose praziquantel 40 mg/kg body weight administered for the treatment of intestinal schistosomiasis among school children living in a rural area along the shore of Lake Victoria in north-western Tanzania. The study area is known to have a high prevalence of schistosomiasis despite repeated praziquantel MDA. Efficacy was assessed using both CR and ERR after a three week follow up period (21 days), as recommended by the WHO [30]. Our results indicate a high CR (81.2%) and ERR (95.0%) of praziquantel against *S. mansoni* infection. A single dose praziquantel treatment resulted in a significant reduction in the infection intensity (Table 3). Overall, a small proportion of infected children (12.3%) remained with light infection, and very few participants (5.6%) with moderate or heavy infections (0.9%) after treatment. This finding indicates that praziquantel reduces schistosomiasis-associated morbidities attributed to moderate and heavy *S. mansoni* infection in children. The observed CR (81.2%) in this study falls within the documented range of 60–90% for *S. mansoni* infection [10]. The WHO guideline for assessing the efficacy of praziquantel against schistosomiasis states that an ERR of >90% is satisfactory [30]. The observed high CR and ERR in this study indicate that praziquantel is still efficacious against this species of *Schistosoma,* and praziquantel can still be used by the national NTD control programs in MDA among school children and other at-risk groups in Tanzania. 

Safety and efficacy surveillance reports are important to inform the WHO and the national NTD control programs on the current status of the drug efficacy for close follow ups and decision making. To date, there is no alternative drug to treat schistosomiasis. Since 1984, praziquantel has been extensively and repeatedly used in large-scale MDA programs for the control and prevention of schistosomiasis. Praziquantel’s low CR may be due to the increased number of MDA rounds [13]. Thus, continuous close monitoring of praziquantel efficacy in endemic countries, particularly in high transmission areas, is vital for the early detection of drug tolerance or resistance. To justify the need for a regular assessment of praziquantel efficacy, a recent study in Cote d’Ivoire reported a much lower CR (69%) at 21–25 days follow up among school children infected with *S. mansoni* [20]. 

Despite the observed higher CR of praziquantel, about one-fifth of the children (18.8%) were not cured of the diseases at three weeks post-treatment in this study. The observed 18.8% non-cure rate in our study cannot be attributed to reinfection, since the stool examination was done at three weeks post-drug administration (21 days), as recommended by the WHO [30]. The poor efficacy of praziquantel against immature parasites has been elucidated as a possible cause of low CRs, especially if the assessment of drug efficacy is done > three weeks post-drug administration particularly high transmission areas, where the residents are continuously exposed for new infections and harbor more immature parasites [16]. A recent study conducted in north-western Tanzania, where the assessment was done eight weeks post-treatment, reported a lower CR, i.e., 68.68% [31]. Similar low CR (60.9%) and ERR (61.4%) were reported from Côte d’Ivoire at six weeks post-treatment [25]. The poor efficacy of praziquantel against juvenile schistosomes is well recognized, and hence, the need for further treatment optimization research is evident. To enhance praziquantel performance, randomized clinical trials (RCTs) to investigate the combination of praziquantel with other drugs that are active against the juvenile worms (such as artemisinins) [32], or the use of repeated praziquantel doses, may be considered in endemic settings [16,31].

On the other hand, praziquantel at a single dose of 40 mg/kg body weight continues to be a safe and tolerable drug among infected school children, as observed in this study. Only 28.7% of the study participants experienced treatment-associated adverse events within four hours of praziquantel administration. Furthermore, in this study, almost all of the observed adverse events among the study participants were mild and transient, similar to reports from other studies [19,20,21,25], and no serious adverse events were reported. Praziquantel is reported to be associated with gastrointestinal symptoms (such as abdominal pain, nausea, vomiting, and diarrhea), neurological symptoms (including headache, muscle pain, drowsiness, dizziness, and fainting), and dermatological symptoms (such as itching, skin rashes) [33]. 

The type and severity of the adverse events mainly depend on pre-treatment infection intensity and feeding status of the children before drug administration. As recommended by the WHO [30], the provision of meals before drug administration may help to reduce the nauseating effect of the drug, and hence, also to reduce the occurrence and severity of treatment-associated adverse events. In this study, abdominal pain was the most observed adverse event (26.7%). A similar finding is reported from other previous studies [19,20,21,25]. The abdominal pain was significantly associated with pre-treatment infection intensity of the study participants, similar to what was reported in other studies [21]. Children with moderate to heavy infections experienced more abdominal pain compared to those with light infections. On the other hand, vomiting was significantly associated with anemia in the studied population. Participants who were anemic experienced vomiting more than those without anemia. This finding is important, especially in settings like the Lake Zone in Tanzania, where a significant percentage of children have been reported to be anemic [7,28]. The national NTD control program should consider assessing children for anemia (e.g., by physical examination) prior to mass praziquantel administration. Our study observed few adverse events (abdominal pain and vomiting only) compared to other studies, where adverse events like drowsiness, dizziness, muscle pain, fatigue, and weakness were also reported [21,22,33]. The difference can be attributed to study population differences, including genetics, feeding status before drug administration, nutritional status, and disease prevalence and intensity in a given region [34,35,36]. In these circumstances, where treatment-associated adverse events are dependent on pre-treatment infection intensity and anemia status, safety monitoring in the mass praziquantel administration to school children in endemic settings remains crucial. 

## 4. Materials and Methods 

### 4.1. Ethical Statement

This study received ethical approval from the Institution Review Board of Muhimbili University of Health and Allied Sciences (Ref: 2016-5-25/AEC/Vol.X/03) and the Medical Research Coordination Committee of National Institute for Medical Research, Tanzania (NIMR/HQ/R.8a/Vol.IX/2343). Before commencement of the study, permission to conduct the study was also granted by relevant government authorities of Busega district including District Executive Director (DED), District Medical Officer (DMO), District Education Officer (DEO), Village Leaders, School board and administration. Written informed consent from parents/guardians and assent from participating children were obtained. 

### 4.2. Study Area

The study was conducted in Nyamikoma village, Busega district, north-western Tanzania between May 2017 and January 2018 in collaboration with the National Institute for Medical Research (NIMR), Mwanza Research Centre Tanzania. Nyamikoma village is located approximately one kilometer from the Lake Victoria basin; it is one of the endemic settings in Busega district. Busega district is one of the administrative districts in Simiyu region; it borders the Lake Victoria to the North. Schistosomiasis is among the top 10 diseases of public health importance in the district. The main economic activities around the district include fishing in Lake Victoria and farming of both animal husbandry and crops. The lake is the main source of water for all human needs in the village and school.

### 4.3. Study Design and Population

This was an observational prospective safety and efficacy surveillance study of praziquantel in *Schistosoma mansoni* infected school children. The efficacy of praziquantel was measured at three weeks post-treatment (21 days) and treatment-associated adverse events were monitored within four hours of post-treatment. A total of 341 school children (aged 7–17 years) infected with intestinal schistosomiasis residing in Nyamikoma village were enrolled in the study. 

### 4.4. Data Collection Procedures

Sociodemographic data including age (school registry), gender, and clinical data such as pre-treatment presenting symptoms, hemoglobin (Hb) concentration, anthropometric measurements (body weight and height), egg count, and observed treatment-associated adverse events from each participant were collected using the case record form prepared for the study.

#### 4.4.1. Stool Sample Examination: Field and Laboratory Procedures

Two fresh stool samples were collected on two consecutive days, both at the baseline survey (pre-treatment) and three weeks post-treatment for screening (diagnosis), and the assessment of drug efficacy (CR and ERR), respectively. Four thick Kato Katz smears were prepared using a 41.7 mg template [37] in a temporary field laboratory by experienced field technicians from NIMR Mwanza Research Centre. The thick Kato Katz smears were transported to NIMR Mwanza Research Centre laboratory and examined by two trained and experienced technicians. To ensure the clear visibility of *Schistosoma* eggs, microscopic examination of the smears was done 24 h after the preparation of the slides to allow adequate coloration of the eggs by the stain (methylene blue). About 10% of the smears were subjected to quality control by an external technician. The mean egg count of each participant was calculated as an average of the four prepared thick Kato Katz slides. *S. mansoni* infection intensity described as eggs per gram of stool (epg) was calculated by multiplying the mean egg count by a constant factor of 24 [38], and classified according to the WHO classification; (i) light infection (epg < 100), (ii) moderate infection (epg 100–399), and (iii) heavy infection (epg ≥ 400) [39]. Examination of the parasitological CR and ERR was conducted three weeks after drug administration (21 days), as recommended by the WHO [30].

#### 4.4.2. Estimation of Hemoglobin Concentration and Anthropometric Measurements

Before praziquantel administration, finger-prick blood (100 µL) was collected to measure hemoglobin concentration (Hb conc) in g/dL using the HemoCue Hb 201+ analyzer (HemoCue AB Angelholm, Sweden) [40]. A child was defined as anemic if the Hb conc was less than 11.5 g/dL [41]. Anthropometric measurements were used to assess the nutritional status of the children. Bodyweight was measured in kilograms (kg), and height was measured in centimeter (cm). Body mass index (BMI) was calculated as a ratio of body weight (in kg) to height square (in meters). To assess for stunting and wasting, the anthropometry were converted to Z scores as height for age Z score (HAZ) and body mass index (BMI) for age Z score (BAZ) using the WHO Anthro-Plus software version 1.0.4 (Department of Nutrition, WHO, Avenue Appie, Geneva, Switzerland) [42]. All children with HAZ and BAZ scores less than two standard deviations were considered stunted and wasted, respectively.

#### 4.4.3. Drug Administration and Safety Assessment

The praziquantel tablets used in this study were obtained as a donation from the Tanzania NTD control program. The Tanzania NTD control program receives praziquantel tablets from the WHO. Before drug administration, study participants were given a standardized meal (porridge and biscuits) to minimize the nauseating effect of praziquantel. Thereafter, a single dose of 40 mg/kg body weight of praziquantel (Batch BZ6043, S Kant Health Care Ltd, India) was administered to each study participant as recommended by the WHO for the treatment of schistosomiasis [30]. Following drug administration, study participants were closely monitored for any treatment-associated adverse events including gastrointestinal, neurological, and dermatological symptoms presenting within four hours of drug administration, as described previously [21,22,23,24].

### 4.5. Study Outcomes

The primary outcome of the study was efficacy (CR and ERR) based on the thick smear Kato-Katz method at three weeks post-treatment. The CR was defined as the proportion of egg positive (infected) children before treatment who became egg negative (eggs free) at three weeks post-treatment. The ERR was calculated as 100 times [1 − (Arithmetic mean of epg after treatment/Arithmetic mean of epg before treatment)], as recommended by the WHO [30]. Secondary outcome was treatment-associated adverse events presented or reported within four hours of drug administration.

### 4.6. Data Management and Statistical Analysis

All data were handled by the Data Management Unit (DMU) of NIMR Mwanza Research Centre. Data were double entered in a database (Census and Survey Processing System (CSPro) software (US Census Bureau, Washington, DC, USA), cleaned, and then exported into an excel file. Data analysis was done using Statistical Package for Social Sciences (SPSS) software for Windows version 20 (SPSS, IBM Corp, Armonk, NY, USA). Descriptive statistics were used to analyze the socio-demographic data, baseline characteristics, and results summarized in frequency tables. Depending on the test appropriateness, a Pearson’s chi-square test or Fisher’s exact test was used to compare proportions between groups. A Mann Whitney U test was used to compare means of the egg count at baseline and at the follow up visit between groups. Univariate and multivariate regression analyses were used to determine the predictors of cure. Variables with p-value < 0.3 on univariate analysis were included in the multivariate regression model. Statistical significance was considered at a *p*-value of < 0.05.

## 5. Conclusions

Praziquantel given as a single dose (40 mg/kg body weight) is still efficacious and safe against *Schistosoma mansoni* infection, as per the WHO guideline. The drug can still be used in MDA by the national NTD programs among school children and other at-risk populations. However, the failure to cure in one-fifth (18.8%) and adverse events in more than a quarter (28.7%) of the infected children indicate the need for regular and close monitoring of the safety and efficacy of the drug, especially in areas of high transmission in endemic settings. Further studies to optimize praziquantel safety and performance are worth exploring to achieve schistosomiasis control and eventual elimination. 

## Figures and Tables

**Table 1:** Socio-demographic and baseline characteristics of the studied population.

Characteristic		N	% (95% CI)
Age (years)	Mean ±SD	11.8 ± 1.7	
	≤12	235	68.9 (64.1–73.6)
	>12	106	31.1 (26.4–35.9)
Sex	Male	160	46.9 (41.8–52.5)
	Female	181	53.1 (47.5–58.2)
Infection intensity	Light	87	25.5 (20.9–30.0)
	Moderate	152	44.6 (39.0–50.0)
	Heavy	102	29.9 (24.9–34.9)
Pre-treatment abdominal pain	Yes	71	20.8 (16.7–25.3)
	No	270	79.2 (74.7–83.2)
Stool consistency	Loose	51	15.0 (11.0–19.0)
	Soft	154	45.2 (39.8–50.1)
	Formed	136	39.9 (34.5–45.2)
Stunting (HAZ)	Stunted	117	34.3 (29.2–39.5)
	Not stunted	224	65.7 (60.2–70.6)
Wasting (BAZ)	Wasted	34	10.0 (6.8–13.2)
	Not wasted	307	90.0 (86.5–93.2)
Hemoglobin concentration	Median (IQR)	12.7 (11.6–13.5)	
Egg count/gram of stool	Mean ±SD	365.1 ± 437.4	
	Median (IQR)	222 (96–471)	

BAZ: body mass index (BMI) for age Z score; HAZ: height for age Z score; epg: egg count/gram of stool; CI: Confidence interval.

**Table 2:** Association of sociodemographic and baseline characteristics with cure rates among study participants.

Variable		Cured N (%)	Not Cured N (%)	*χ^2^* Value	*p*-Value
Age group	≤12	190 (80.9)	45 (19.1)	0.072	0.79
	>12	87 (82.1)	19 (17.9)		
Sex	Male	128 (80.0)	32 (20.0)	0.300	0.58
	Female	149 (82.3)	32 (17.7)		
Infection intensity	Light	72 (82.8)	15 (17.2)	1.365	0.51
	Moderate	126 (82.9)	26 (17.1)		
	Heavy	79 (77.5)	23 (22.5)		
Stunting (HAZ)	Stunted	98 (83.8)	19 (16.2)	0.747	0.39
	Not stunted	179 (79.9)	45 (20.1)		
Wasting (BAZ)	Wasted	30 (88.2)	4 (11.8)	1.215	0.27
	Not wasted	247 (80.5)	60 (19.5)		
Anemia status	Anemic	68 (88.3)	9 (11.7)	3.270	0.07
	Not anemic	209 (79.2)	55 (20.2)		
Stool consistency	Loose	40 (78.4)	11 (21.6)	0.726	0.70
	Soft	128 (83.1)	26 (16.9)		
	Formed	109 (80.1)	27 (19.9)		

**Table 3:** Proportion of infection intensity before and after a single-dose praziquantel treatment among all study participants (n = 341), and treatment response stratified by pretreatment infection intensity.

Overall Infection Intensity		Before Treatment N (%)	After Treatment N (%)
Light		87 (25.5)	42 (12.3)
Moderate		152 (44.6)	19 (5.6)
Heavy		102 (29.9)	3 (0.9)
Cured		-	277 (81.2)
**Proportion of cured and uncured children within each infection intensity group before and after treatment**			
**Before treatment**		**After treatment**	
**Status**	**N (%)**	**Status**	**N (%)**
Light infection	87 (25.5)	Cured	72 (82.8)
		Light	13 (14.9)
		Moderate	2 (2.3)
		Heavy	0 (0.0)
Moderate infection	152 (44.6)	Cured	126 (82.9)
		Light	16 (10.5)
		Moderate	9 (5.9)
		Heavy	1 (0.7)
Heavy infection	102 (29.9)	Cured	79 (77.5)
		Light	13 (12.5)
		Moderate	8 (7.8)
		Heavy	2 (2.0)

**Table 4:** Predictors of cure at week three post single-dose praziquantel treatment.

Variable	Categories	Cured N (%)	Univariate Analysis			Multivariate Analysis		
			cOR	95% CI	*p*-Value	aOR	95% CI	*p*-Value
Age			0.91	0.78–1.07	0.26	0.96	0.76–1.22	0.76
Sex	Male	128 (80.0)	0.86	0.49–1.48	0.58			
	Female	149 (82.3)	1^a^					
Log Baseline egg count			1.28	0.77–2.15	0.34			
Anemia	Anemic	68 (88.3)	1.99	0.93–4.24	0.07	0.48	0.22–1.04	0.06
	Not anemic	209 (79.2)	1^a^					
Log Hb conc			3.45	0.07–179.99	0.54			
Log weight			0.26	0.01–6.00	0.40			
Log height			0.01	0.001–4.77	0.24	0.004	0.004–1796.26	0.41
Wasting (BAZ)	Wasted	30 (88.2)	1.13	0.85–1.51	0.39			
	Not wasted	247 (80.5)	1^a^					
Stunting (HAZ)	Stunted	98 (83.8)	0.98	0.75–1.26	0.85			
	Not stunted	179 (79.9)	1^a^					
Baseline infection intensity	Light	72 (82.8)	1.41	0.75–2.64	0.28	1.44	0.76–2.73	0.26
	Moderate	126 (82.9)	1.01	0.50–2.03	0.97	0.99	0.49–2.02	0.99
	Heavy	79 (77.5)	1^a^					

N: Total number of participants within each category; 1^a^: reference category; aOR: adjusted odds ratio; cOR: crude odds ratio.

**Table 5:** The Arithmetic mean egg count at baseline and follow up and their respective egg reduction rates by age group and sex.

Variable		Baseline Egg Count (Mean ± SD)	*p*-Value ^¥^	Follow up Egg Count (Mean ± SD)	*p*-Value ^¥^	ERR * (%)
Age group	≤12 years	341.9 ± 433.0	0.07	21.7±74.8	0.64	93.6
	>12years	416.3 ± 444.8		10.2±36.8		97.6
Sex	Male	379.6 ± 405.3	0.19	15.5±53.5	0.65	96.0
	Female	352.2 ± 464.7		20.6±74.7		94.2

* ERR = 100 [1 − (Arithmetic mean epg after treatment/Arithmetic mean epg before treatment)], ^¥^: Mann Whitney U test.

**Table 6:** Association between socio-demographic and baseline characteristics and observed adverse events among study participants.

Variable	Abdominal Pain				Vomiting			
	Yes N (%)	No N (%)	*χ^2^* Value	*p*-Value	Yes N (%)	No N (%)	*χ^2^* Value	*p*-Value
Age (years)								
≤12	68 (28.9)	167 (71.1)	1.956	0.16	6 (2.6)	229 (97.4)		0.18 ^β^
>12	23 (21.7)	83 (78.3)			0 (0.0)	106 (100)		
Sex								
Male	38 (23.8)	122 (76.2)	1.328	0.25	2 (1.2)	158 (98.8)		0.69 ^β^
Female	53 (29.3)	128 (70.7)			4 (2.2)	171 (97.8)		
Stunting (HAZ)								
Stunted	29 (24.8)	88 (75.2)	0.329	0.57	3 (2.6)	114 (97.4)		0.42 ^β^
Not stunted	62 (27.7)	162 (72.3)			3 (1.3)	221 (98.7)		
Wasting (BAZ)								
Wasted	8 (23.5)	26 (76.5)	0.192	0.66	1 (2.9)	33 (97.1)		0.47 ^β^
Not wasted	83 (27.0)	224 (73.0)			5 (1.6)	302 (98.4)		
Anemia status								
Anemic	26 (33.8)	51 (66.2)	2.548	0.11	4 (5.2)	73 (94.8)		0.03 ^β^
Not anemic	65 (24.6)	199 (75.4)			2 (0.8)	262 (99.2)		
Infection intensity								
Light	11 (12.6)	76 (87.4)	18.366	<0.001	1 (1.1)	86 (98.9)	1.184	0.55
Moderate	39 (25.7)	113 (74.3)			2 (1.3)	150 (98.7)		
Heavy	41 (40.2)	61 (59.8)			3 (2.9)	99 (97.1)		

^β^ Fishers exact test.
